# Peroxisome proliferator-activated receptor-α (PPARα) regulates wound healing and mitochondrial metabolism in the cornea

**DOI:** 10.1073/pnas.2217576120

**Published:** 2023-03-21

**Authors:** Wentao Liang, Li Huang, Amy Whelchel, Tian Yuan, Xiang Ma, Rui Cheng, Yusuke Takahashi, Dimitrios Karamichos, Jian-Xing Ma

**Affiliations:** ^a^Department of Physiology, University of Oklahoma Health Sciences Center, Oklahoma City, OK 73104; ^b^Department of Biochemistry, Wake Forest University School of Medicine, Winston-Salem, NC 27157; ^c^Department of Ophthalmology, Fujian Medical University Union Hospital, Fuzhou 350000, China; ^d^Division of Research and Innovation, North Texas Eye Research Institute, University of North Texas Health Science Center, Fort Worth, TX 76107; ^e^Department of Pharmaceutical Sciences, University of North Texas Health Science Center, Fort Worth, TX 76107; ^f^Department of Pharmacology and Neuroscience, University of North Texas Health Science Center, Fort Worth, TX 76107

**Keywords:** cornea, epithelium, wound healing, PPARα, mitochondria

## Abstract

Diabetes can result in impaired corneal wound healing. Mitochondrial dysfunction plays an important role in diabetic complications. However, the regulation of mitochondria function in the diabetic cornea and its impacts on wound healing remain elusive. The present study aimed to explore the molecular basis for the disturbed mitochondrial metabolism and subsequent wound healing impairment in the diabetic cornea. Seahorse analysis showed that mitochondrial oxidative phosphorylation is a major source of ATP production in human corneal epithelial cells. Live corneal biopsy punches from type 1 and type 2 diabetic mouse models showed impaired mitochondrial functions, correlating with impaired corneal wound healing, compared to nondiabetic controls. To approach the molecular basis for the impaired mitochondrial function, we found that Peroxisome Proliferator-Activated Receptor-α (PPARα) expression was downregulated in diabetic human corneas. Even without diabetes, global *PPARα* knockout mice and corneal epithelium-specific *PPARα* conditional knockout mice showed disturbed mitochondrial function and delayed wound healing in the cornea, similar to that in diabetic corneas. In contrast, fenofibrate, a PPARα agonist, ameliorated mitochondrial dysfunction and enhanced wound healing in the corneas of diabetic mice. Similarly, corneal epithelium-specific *PPARα* transgenic overexpression improved mitochondrial function and enhanced wound healing in the cornea. Furthermore, PPARα agonist ameliorated the mitochondrial dysfunction in primary human corneal epithelial cells exposed to diabetic stressors, which was impeded by siRNA knockdown of *PPARα*, suggesting a PPARα-dependent mechanism. These findings suggest that downregulation of PPARα plays an important role in the impaired mitochondrial function in the corneal epithelium and delayed corneal wound healing in diabetes.

## Significance

Diabetic keratopathy (DK) is a common diabetic complication and can impair corneal wound healing. DK lacks effective therapy as its pathogenesis is unclear. In this work, we identified for the first time that human corneal epithelial cells utilize mitochondrial oxidative phosphorylation as a major source of ATP production. In addition, we identified PPARα as a key regulator of mitochondrial function in the cornea and found that diabetes-induced PPARα downregulation plays a pathogenic role in mitochondrial dysfunction and impaired wound healing in the cornea. These findings identified a new function of PPARα in the cornea and revealed a new pathogenic mechanism for diabetes-induced corneal wound healing delay. Further, these results suggest that PPARα agonist has therapeutic potential for DK.

Diabetes mellitus (DM) is a metabolic disease, which can lead to a number of complications including chronic kidney disease, nerve damage, and other problems with feet, vision, and mental health (1–5). Diabetic corneal complications including corneal nerve degeneration, wound healing delay, and ulcer occur in more than half of diabetic population (6–8). Despite extensive research, the diabetic cornea lacks effective treatment, and the pathogenesis for the corneal defects induced by diabetes is not fully understood.

Growing evidence has shown that mitochondrial dysfunction is closely associated with diabetic complications (9–11). However, the direct impact of diabetes on corneal metabolism is unclear, representing a significant knowledge gap. The metabolic profiles of corneal cells in diabetes and their regulation have not been previously investigated. The molecular basis underlying impaired wound healing in the diabetic cornea has not been well understood.

Peroxisome Proliferator-Activated Receptor-α (PPARα) was originally known to regulate lipid metabolism in the liver (12). Its agonists are clinically used for the treatment of dyslipidemia (13–15). More recently, PPARα agonist fenofibrate has been reported to have a robust therapeutic effect on diabetic retinopathy in type 2 diabetic patients (16, 17). Our recent study demonstrated, for the first time, that PPARα is expressed at high levels in the cornea, particularly in epithelial cells, and *PPARα* knockout (KO) alone resulted in spontaneous corneal nerve degeneration, epithelial erosion, and impaired corneal sensitivity, closely recapitulating pathologies seen in diabetic keratopathy (DK) in diabetic patients (18). In addition, our previous study showed that PPARα is critical for neuronal survival and energy metabolism in the retina (19). Despite these findings, the role of PPARα in the regulation of corneal cell metabolism and corneal wound healing has not yet been explored.

In the present study, we compared the metabolic profiles of primary human corneal stromal fibroblasts and epithelial cells utilizing a Seahorse XFe96 Analyzer. We determined the role of PPARα in the regulation of mitochondrial function and wound healing in the cornea using *PPARα^−/−^* mice, corneal epithelium-specific *PPARα* conditional KO mice, and transgenic mice. The present study also explored the therapeutic potential of PPARα agonist on wound healing deficiency in the diabetic cornea.

## Results

### Distinct Metabolic Profiles of Human Corneal Epithelial Cells and Stromal Fibroblasts.

**Fig. 1.**
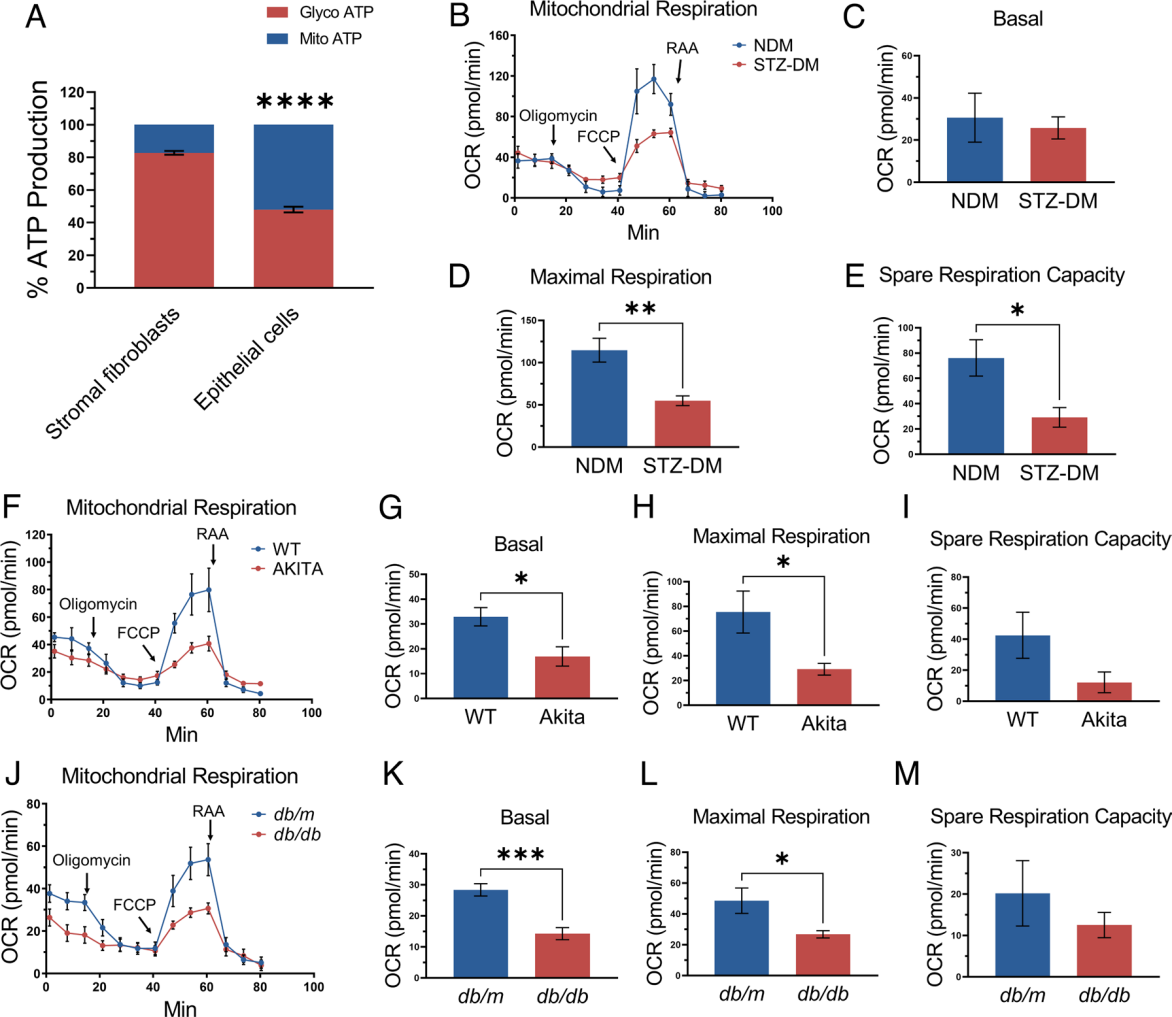
Mitochondrial metabolism is important in the corneal epithelium and is impaired under diabetic condition. (*A*) Real-time ATP rate assay in primary human corneal stromal fibroblasts and epithelial cells (*n* = 6 donors). In stromal fibroblasts, 82.7 ± 1.2% ATP was generated from glycolysis, while only an approximate 17.3 ± 1.2% of total ATP was produced from oxidative phosphorylation. Corneal epithelial cells have approximately 52.0 ± 1.7% of total ATP production originated from oxidative phosphorylation, with 48.0 ± 1.7% from glycolysis. (*B–M*) Mitochondrial stress test in the corneal biopsies from STZ-DM mice (*n* = 6) (*B–E*), Akita mice (*n* = 6) (*F–I*), *db/db* mice (*n* = 8) (*J–M*), and their respective NDM controls. All values are mean ± SEM. *P < 0.05; **P < 0.01; ***P < 0.001; ****P < 0.0001.

To compare the metabolic profiles of human corneal epithelial cells and stromal fibroblasts, adenosine triphosphate (ATP) production was quantified through real-time measurements of the activity of two main ATP-producing pathways, glycolysis and mitochondrial oxidative phosphorylation in primary human cells. To exclude individual variation of different donors, primary corneal epithelial cells and stromal fibroblasts from six human donors were used to measure ATP production individually, and results were averaged. Seahorse analysis showed that 82.7 ± 1.2% ATP was generated from glycolysis, while only an approximate 17.3 ± 1.2% of total ATP was produced by oxidative phosphorylation in stromal fibroblasts, indicating that stromal fibroblasts strongly favored the glycolysis pathway for energy generation. In contrast, corneal epithelial cells have higher utilization of oxidative phosphorylation as energy source, as approximately 52.0 ± 1.7% of total ATP was generated by oxidative phosphorylation, while 48.0 ± 1.7% from glycolysis (Fig. 1*A*). These data suggested that mitochondrial oxidative phosphorylation is an important source of ATP production in the corneal epithelium which is more susceptible to stress by hypoxia and diabetes.

### Impaired Mitochondrial Metabolism in Diabetic Mouse Cornea.

To elucidate mitochondrial function changes in diabetic corneas, we measured the oxygen consumption rate (OCR) using live corneal biopsy punches from type 1 diabetic mouse models [Streptozotocin (STZ)-DM and Akita mice] and a type 2 diabetic mouse model (*db/db* mice) as well as their respective nondiabetic controls. Compared to age-matched nondiabetic controls, STZ-DM mice had depressed maximal respiration and spare respiration capabilities in the cornea (Fig. 1 *B*–*E*). Corneas from Akita mice (16-wk-old) and *db/db* mice (16-wk-old) showed lower basal and maximal OCR than their respective nondiabetic controls (Fig. 1 *F*–*M*). These results suggested that diabetes impaired mitochondrial function in the cornea.

### Decreased PPARα Expression in Diabetic Human Corneas.

**Fig. 2.**
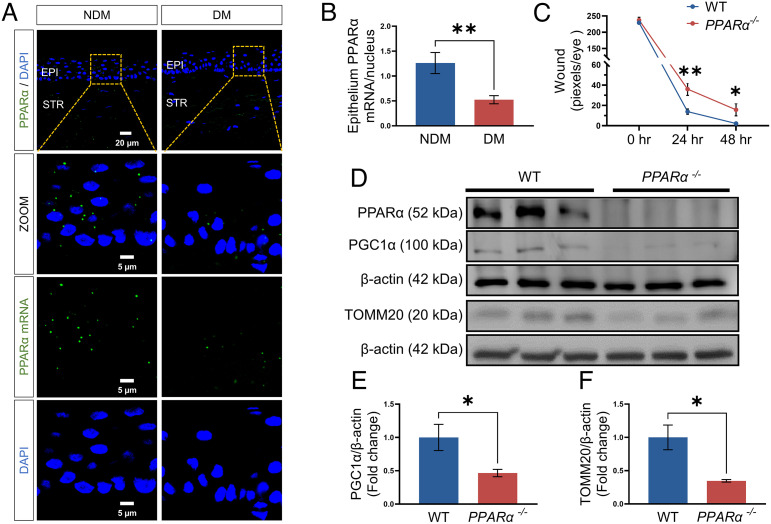
Decreased PPARα levels in diabetic human corneas. (*A*) Representative images of the PPARα mRNA (green) using RNAscope fluorescent assay, with the nuclei counterstained with DAPI (blue) in corneal sections from donors with NDM and DM. EPI, epithelium; STR, stroma. The scale bars represent 20 μm for the upper image and 5 μm for the bottom three images. (*B*) The total PPARα mRNA puncta in the corneal epithelium were counted by ImageJ and normalized by the number of nuclei to quantify the expression of the PPARα mRNA (*n* of NDM = 11, *n* of DM = 13). (*C*) Wound healing in the corneas from *PPARα^−/−^* and WT mice was quantified after fluorescein staining using the pixel per eye with ImageJ (*n* = 10). (*D–F)* Representative Western blots for PPARα, PGC-1α, TOMM20, and β-actin and densitometry quantification in the corneas from 5-mo-old *PPARα^−/−^* mice and WT littermates (*n* = 3). All values are mean ± SEM. *P < 0.05; **P < 0.01.

Previously, we reported that PPARα is expressed in the cornea, and its protein levels are decreased in the corneas of diabetic patients and rats (18). Here, the RNAscope assay was used to measure PPARα mRNA levels in the cornea from human donors with diabetes (13 DM, 2 females and 11 males, the average age was 68.3 y) and without diabetes (11 NDM, five females and six males, the average age was 63.5 y). The result showed that the expression of the PPARα mRNA was downregulated in the corneal epithelium of human donors with DM relative to the nondiabetic corneas (Fig. 2 *A* and *B*).

### Impaired Wound Healing and Decreased Mitochondrial Contents in the Cornea of PPARα^−/−^ Mice.

To explore the role of PPARα in corneal wound healing and mitochondrial function, we induced corneal epithelial wound in *PPARα^−/−^* mice and their wild-type (WT) littermates. The corneal wound healing was significantly slower in *PPARα^−/−^* mice than that in WT mice (Fig. 2*C*). Western blot analysis showed that levels of mitochondrial marker Translocase of Outer Mitochondrial Membrane 20 (TOMM20) were significantly reduced in the cornea of *PPARα^−/−^* mice at 5 mo of age, relative to their WT littermates (Fig. 2 *D* and *E*). In addition, PGC-1α levels in the corneas of *PPARα^−/−^* mice were significantly lower than those of WT controls (Fig. 2 *D* and *F*). The results suggested that the ablation of *PPARα* alone resulted in decreased mitochondrial biogenesis and impaired wound healing in the cornea, similar to the phenotypes observed in diabetic mice.

### Activation of PPARα by Fenofibrate Ameliorated Corneal Wound Healing Delay in STZ-DM Mice.

**Fig. 3.**
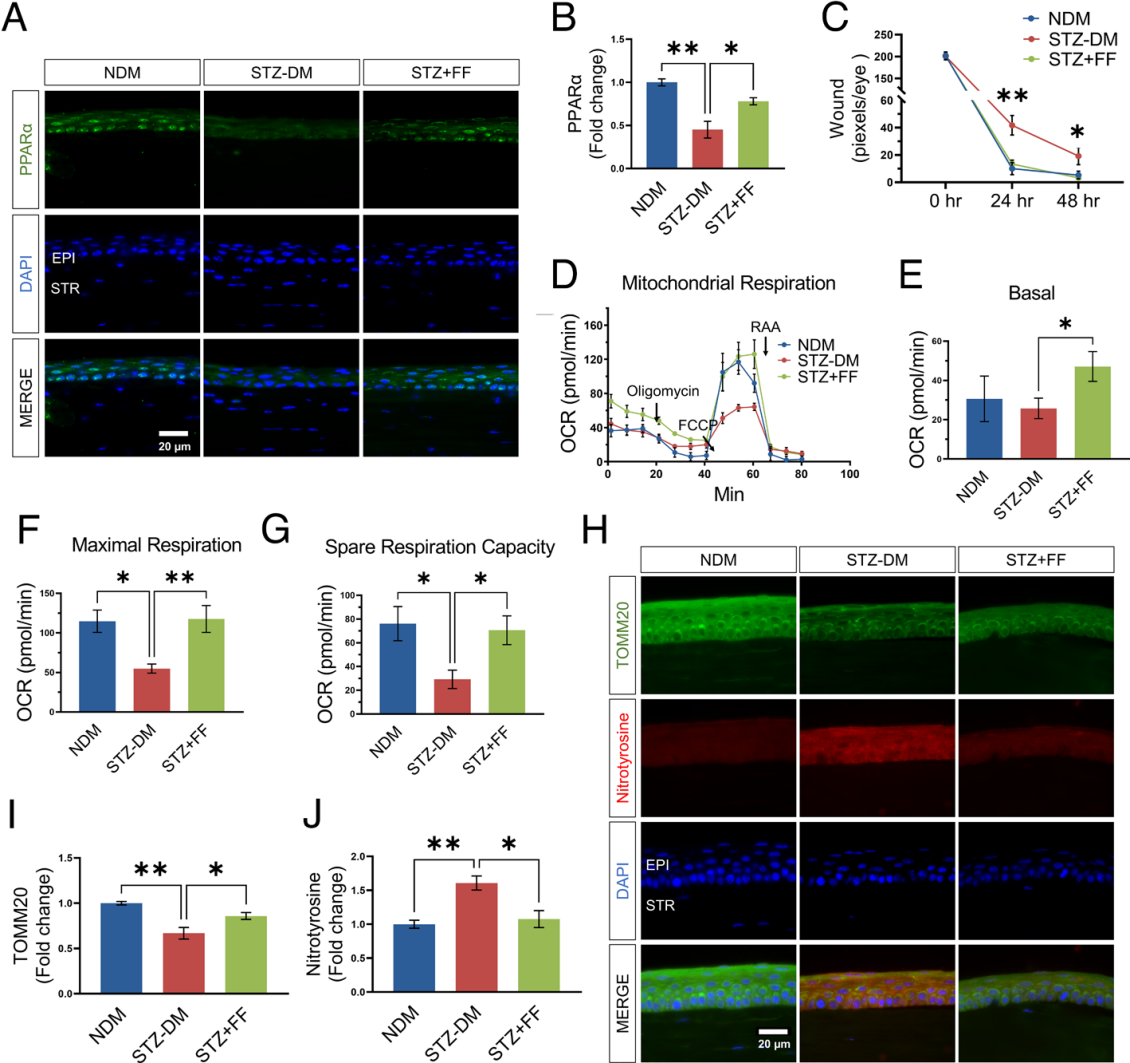
Fenofibrate treatment promoted corneal PPARα expression and prevented mitochondrial function decline in the cornea of STZ-DM mice. (*A*) Representative immunohistochemistry images with an antibody against PPARα (green) in the cornea from nondiabetic mice (NDM), STZ-induced diabetic mice (STZ-DM, 3 mo of diabetes) fed with regular chow or chow containing 0.014% fenofibrate (STZ+FF). (Scale bars, 20 µm.) (*B*) The intensity of PPARα signals in the epithelial layer in (*A*) was quantified using ImageJ (*n* = 3). (*C*) Quantification of the corneal wound. The progress of wound healing was quantified by measuring wound severity using the pixel of green fluorescence per eye with Image J (*n* =16). (*D–G*) Mitochondrial stress test in the corneas from STZ-induced DM mice and NDM controls fed with regular chow or chow containing fenofibrate (*n* = 6). (*H*) Representative immunohistochemistry staining images of TOMM20 (green) and nitrotyrosine (red) with the nuclei counterstained by DAPI (blue) in the corneas from NDM mice, STZ-induced diabetic mice with normal chow or fenofibrate chow. *I&J*: Immunostaining intensities of TOMM20 (*I*) and nitrotyrosine (*J*) in the epithelial layer in (*H*) were quantified using ImageJ (*n* = 3). All values are mean ± SEM. *P < 0.05; **P < 0.01.

Other groups and we demonstrated deficient corneal epithelial wound healing in diabetic animals (20–24). To explore the role of PPARα in diabetic corneal wound healing, we fed STZ-induced diabetic (STZ-DM) C57BL/6J mice with fenofibrate chow for 3 mo starting at the diabetes onset. As shown in Fig. 3 *A*–*C*, fenofibrate significantly rescued the expression level of epithelial PPARα and ameliorated the corneal wound healing delay in STZ-DM mice.

### Fenofibrate Prevented Corneal Mitochondrial Dysfunction in STZ-DM Mice.

To investigate whether activation of PPARα alleviates metabolic deficiency in diabetic corneas, we measured the real-time mitochondrial respiration using live corneal biopsy punches. The results showed that maximal respiratory rate and spare respiratory capacity were significantly decreased in the corneas of STZ-DM mice, compared to those in age-matched nondiabetic mice. Fenofibrate treatment attenuated the decreases of the basal and maximal respiratory rate and spare respiratory capacity in diabetic mice (Fig. 3 *D*–*G*). To further investigate whether fenofibrate also regulates mitochondria content, we measured corneal levels of a mitochondrial protein TOMM20. As shown by immunostaining, TOMM20 levels were significantly reduced in the cornea of STZ-DM mice, relative to the nondiabetic controls, and greatly restored by fenofibrate chow (Fig. 3 *H* and *I*). Increased contents of nitrotyrosine, a marker of reactive oxygen species, were observed in the cornea of STZ-DM mice, which was dramatically attenuated by fenofibrate chow (Fig. 3 *H* and *J*). Taken together, these results indicated that PPARα activation protects corneal mitochondrial function and increases mitochondrion content in diabetic conditions.

### Corneal Epithelium-Specific PPARα KO Aggravated Mitochondrial Dysfunction and Corneal Wound Healing.

**Fig. 4.**
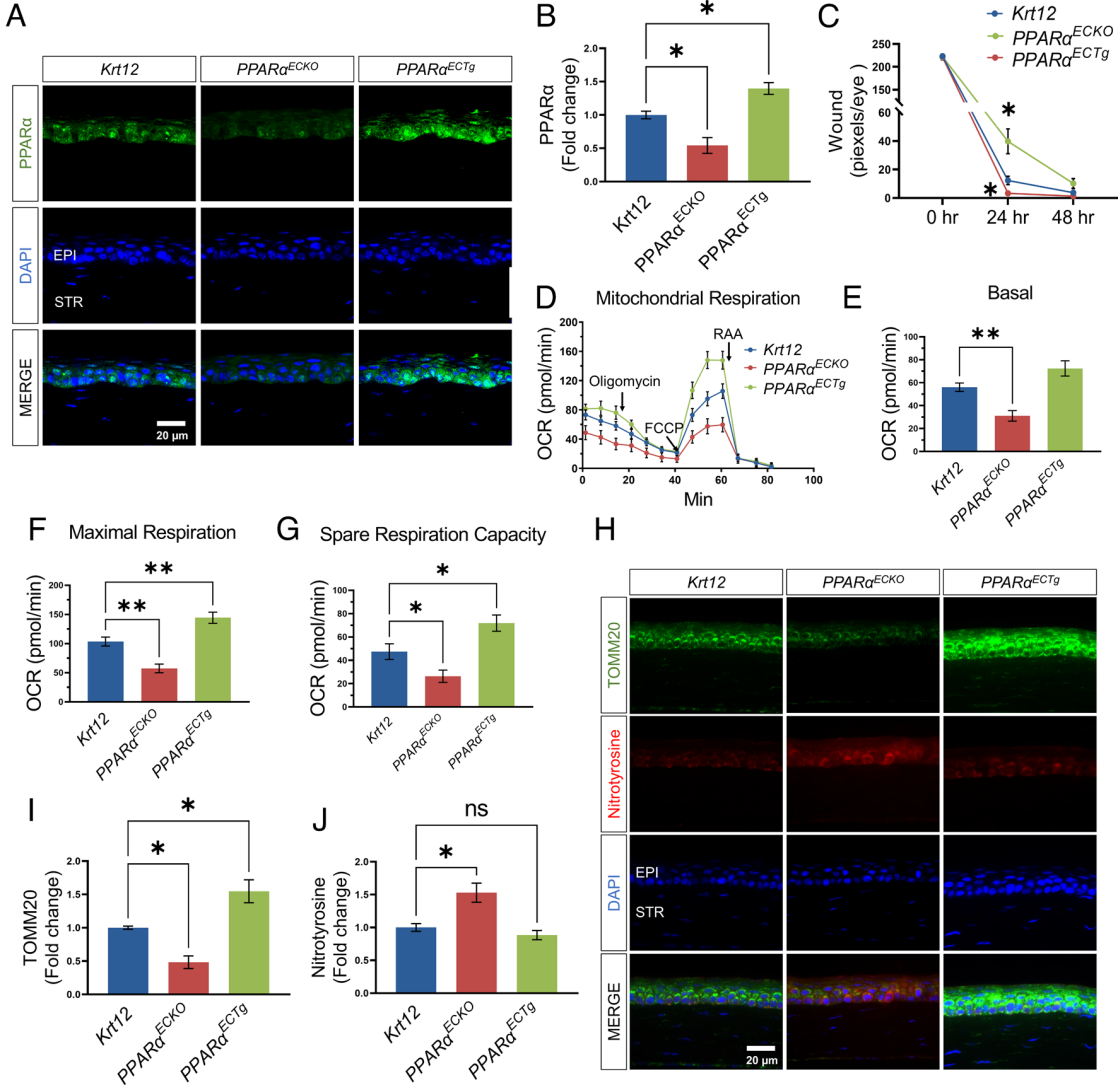
Corneal epithelium-specific *PPARα* KO impaired mitochondrial function and delayed corneal wound healing. (*A*) Representative immunohistochemistry images for PPARα staining (green) in the corneas from *Krt12-Cre (Krt12)PPARα^ECKO^*, and *PPARα^ECTg^* mice. (*B*) The intensity of PPARα in the epithelial layer in (*A*) was quantified using ImageJ (*n* = 3). (*C*) The corneal wound was quantified after fluorescein staining in *Krt12-CrePPARα^ECKO^* and *PPARα^ECTg^* mice and expressed by the pixel per eye with ImageJ (*n* = 12 to 14). (*D–G*) Mitochondrial stress test in the corneas from *Krt12-CrePPARα^ECKO^*, and *PPARα^ECTg^* mice (*n* = 10). (*H*) Representative immunohistochemistry images of TOMM20 (green) and nitrotyrosine (red) in the corneas from *Krt12-CrePPARα^ECKO^*, and *PPARα^ECTg^* mice. *I&J*: The intensities of TOMM20 (*I*) and nitrotyrosine (*J*) in the epithelial layer in (*H*) were quantified using ImageJ (*n* = 3). Values are mean ± SEM. *P < 0.05; **P < 0.01; ns, nonsignificant.

To exclude possible secondary effects of systemic hyperlipidemia in *PPARα^−/−^* mice and further substantiate that mitochondria function is regulated by PPARα in the corneal epithelium, we next used a genetic approach. Corneal epithelium-specific *PPARα* KO (*PPARα^ECKO^*) mice were generated by crossbreeding *PPARα^ flox/flox^* mice with corneal epithelium-specific Cre transgenic (*Krt12-Cre*) mice. To achieve corneal epithelium-specific *PPARα* overexpression, the *PPARα* transgene under the chicken β-actin promoter, which is controlled by a floxed stop cassette, was activated by crossbreeding with *Krt12-Cre* mice to generate *PPARα^ECTg^* mice. As shown by immunostaining, PPARα expression was suppressed in the corneal epithelium of *PPARα^ECKO^* mice and enhanced in the corneal epithelium of *PPARα^ECTg^* mice (Fig. 4 *A* and *B*). *PPARα^ECKO^* mice showed delayed corneal wound healing compared to age-matched *Krt12-Cre* littermates, while *PPARα^ECTg^* mice had accelerated wound healing (Fig. 4*C*).

To evaluate the role of PPARα expression in mitochondrial function in the corneal epithelium, corneal biopsy punches (1.5-mm diameter) from these mice were used for Seahorse analysis. The mitochondrial respiratory function was significantly reduced in the cornea of *PPARα^ECKO^* mice. PPARα overexpression in the corneal epithelium in *PPARα^ECTg^* mice greatly promoted the maximal respiration and spare respiratory capacity, compared to *Krt12-Cre* control mice (Fig. 4 *D*–*G*). Immunostaining showed that TOMM20 levels were significantly reduced in the cornea of *PPARα^ECKO^* mice and greatly elevated in the cornea of *PPARα^ECTg^* mice, relative to the *Krt12-Cre* controls (Fig. 4 *H* and *I*). In addition, nitrotyrosine levels were significantly higher in the corneas of *PPARα^ECKO^* mice than those in *Krt12-Cre* controls (Fig. 4 *H* and *J*).

### PPARα Agonist Treatment Ameliorated the Mitochondrial Integrity Damage Induced by Diabetic Stressors in Primary Human Corneal Epithelial Cells (HCEC).

**Fig. 5.**
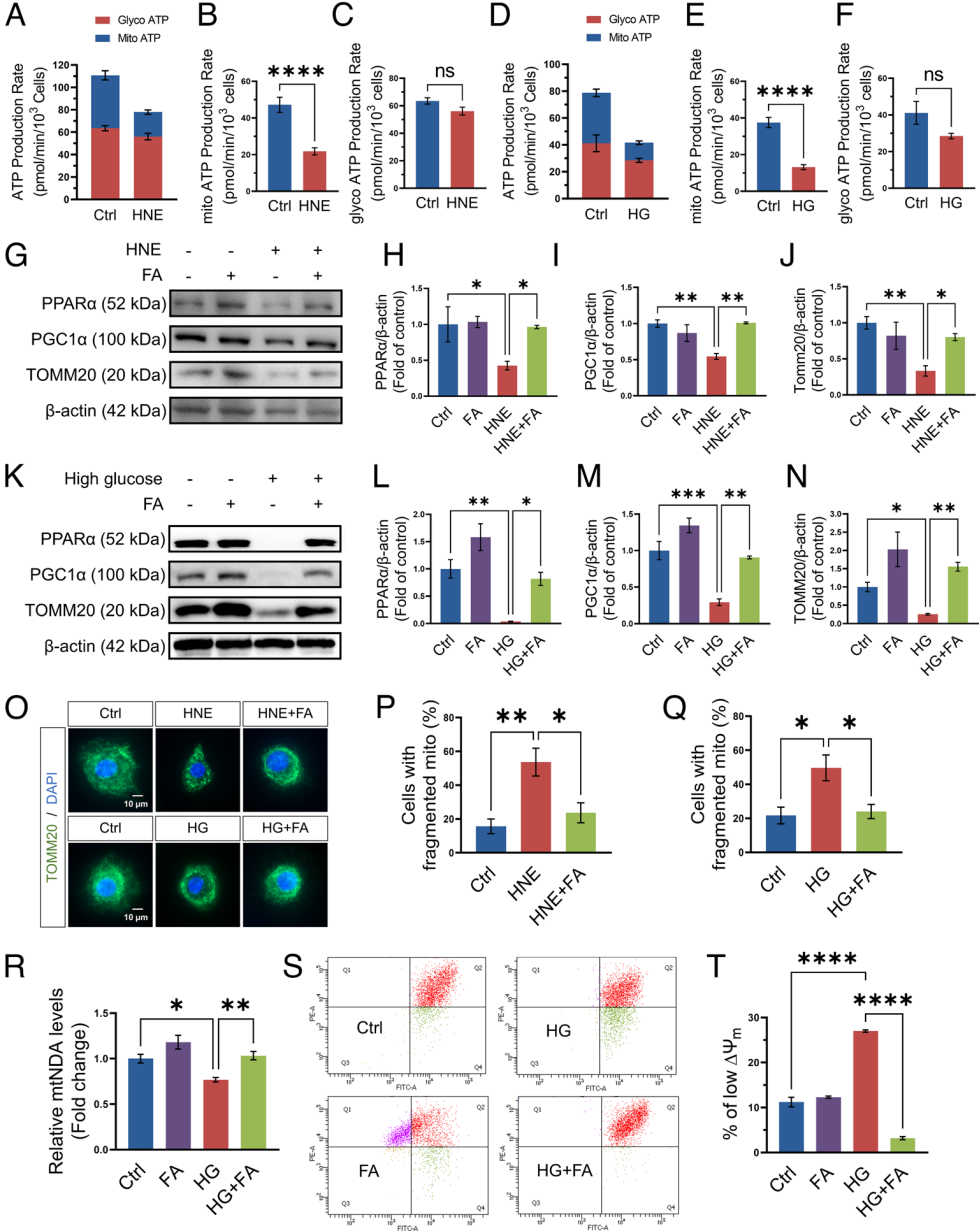
Fenofibric acid treatment ameliorated the impaired mitochondrial integrity induced by diabetic stressor in primary HCEC. (*A*) ATP production from mitochondria and glycolysis was measured using Real-time ATP rate assay in HCEC in the presence or absence of HNE (20 µM) for 24 h (*n* = 9). Vehicle (EtOH)-treated cells were used as control (Ctrl). *B&C*: Comparison of mitochondrial ATP production rate (*B*) and glycolysis ATP production rate (*C*) in panel *A* (*n* = 9). (*D*) ATP production from mitochondria and glycolysis was measured in HCEC exposed to high glucose (HG, 30 mM) for 4 d (*n* = 6). L-glucose-treated cells (6.2 mM D-glucose and 23.8 mM L-glucose) were used as control (Ctrl). *E&F*: Comparison of mitochondrial ATP production rate (*E*) and glycolysis ATP production rate (*F*) in panel *D* (*n* = 6). (*G*) Representative Western blots of PPARα, PGC-1α, TOMM20, and β-actin in HCEC exposed to HNE (20 µM) and fenofibric acid (FA, 20 µM) for 24 h. (*H–J*) PPARα (*H*), PGC-1α (*I*), and TOMM20 (*J*) in (*G*) were quantified by densitometry using ImageJ and normalized by β-actin levels (*n* = 3). (*K*) Representative Western blots of PPARα, PGC-1α, TOMM20, and β-actin in HCEC exposed to high glucose (30 mM) and FA (20 µM) for 4 d. *L-N*: PPARα (*L*), PGC-1α (*M*), and TOMM20 (*N*) in (*K*) were quantified (*n* = 3). (*O*) Representative fluorescence images showing mitochondrial morphology by immunostaining for TOMM20 (green) with the nuclei counterstained by DAPI (blue) in HCEC exposed to HNE (20 µM) or high glucose (30 mM) and FA (20 µM). (*P–Q*) The percentage of cells displaying a fragmented mitochondria was scored under each condition and averaged in three independent experiments. (*R*) Relative mtDNA levels in HCEC treated with high glucose (30 mM) and FA (20 µM) for 4 d (*n* = 5). mtDNA content was normalized to nuclear DNA levels. (*S*) Representative flow cytometry analysis plots showed the distribution of JC-1 stained HCEC exposed to high glucose and FA. (*T*) The percentage of cells with low *∆Ψ*_m_ (Q4) was compared between the groups as indicated (*n* = 3). Values are mean ± SEM. *P < 0.05; **P < 0.01; ***P < 0.001; ****P < 0.0001; ns, nonsignificant.

To establish the direct effect of PPARα signaling on epithelial cells, we used primary human corneal epithelial cells. 4-Hydroxy-2-nonenal (HNE) is a product of lipid peroxidation and a commonly used stressor of diabetes in vitro assays (25–27). HCEC were also stressed with 30 mM D-glucose (with L-glucose as control) to simulate diabetes stress. Real-Time ATP Rate Assay showed that HNE and high glucose both decreased ATP production from mitochondria in HCEC (Fig. 5 *A*–*F*).

As shown by Western blot analysis, HNE or high glucose exposure decreased the expression of PPARα, PGC-1α, as well as TOMM20. Fenofibric acid, an active metabolite of fenofibrate, significantly attenuated the HNE/high-glucose–induced downregulation of PPARα, PGC-1α, and TOMM20 (Fig. 5 *G*–*N*). TOMM20 immunostaining showed that HNE and high glucose both increased HCEC with “fragmented” mitochondria, relative to that in controls. Fenofibric acid significantly decreased HNE/high-glucose–induced mitochondrial fragmentation in HCEC (Fig. 5 *O*–*Q*).

The mitochondrial DNA (mtDNA) copy number is often used as an indicator of mitochondrial mass (28, 29). The qPCR results demonstrated a significant reduction of mtDNA in HCEC exposed to high glucose, which can be prevented by fenofibric acid (Fig. 5*R*).

The mitochondrial membrane electric potential (Δψ_m_) of HCEC was evaluated by JC-1 staining and flow cytometry. In healthy cells with a normal Δψ_m_, the JC-1 dye accumulates in the mitochondria and generates red fluorescence (Q1). In unhealthy cells, the JC-1 enters the mitochondria in a lesser degree and retains the original green fluorescence (Q4) (30). Here, our flow cytometry analysis showed that fenofibric acid treatment group had a higher percentage of Q1 cells, relative to control group (Fig. 5*S*), suggesting that fenofibric acid preserved mitochondrial function in HCEC. After exposure to high glucose, the percentage of low ∆ψ_m_ (Q4) in HCEC was increased compared to the control. Fenofibric acid treatment decreased cells with the low ∆ψ_m_ under high-glucose stress (Fig. 5 *S*–*T*), suggesting that PPARα activation protected mitochondrial integrity.

### Fenofibric Acid Treatment Ameliorated the Mitochondrial Dysfunction Induced by Diabetic Stressors and Enhanced Proliferation and Migration in HCEC.

**Fig. 6.**
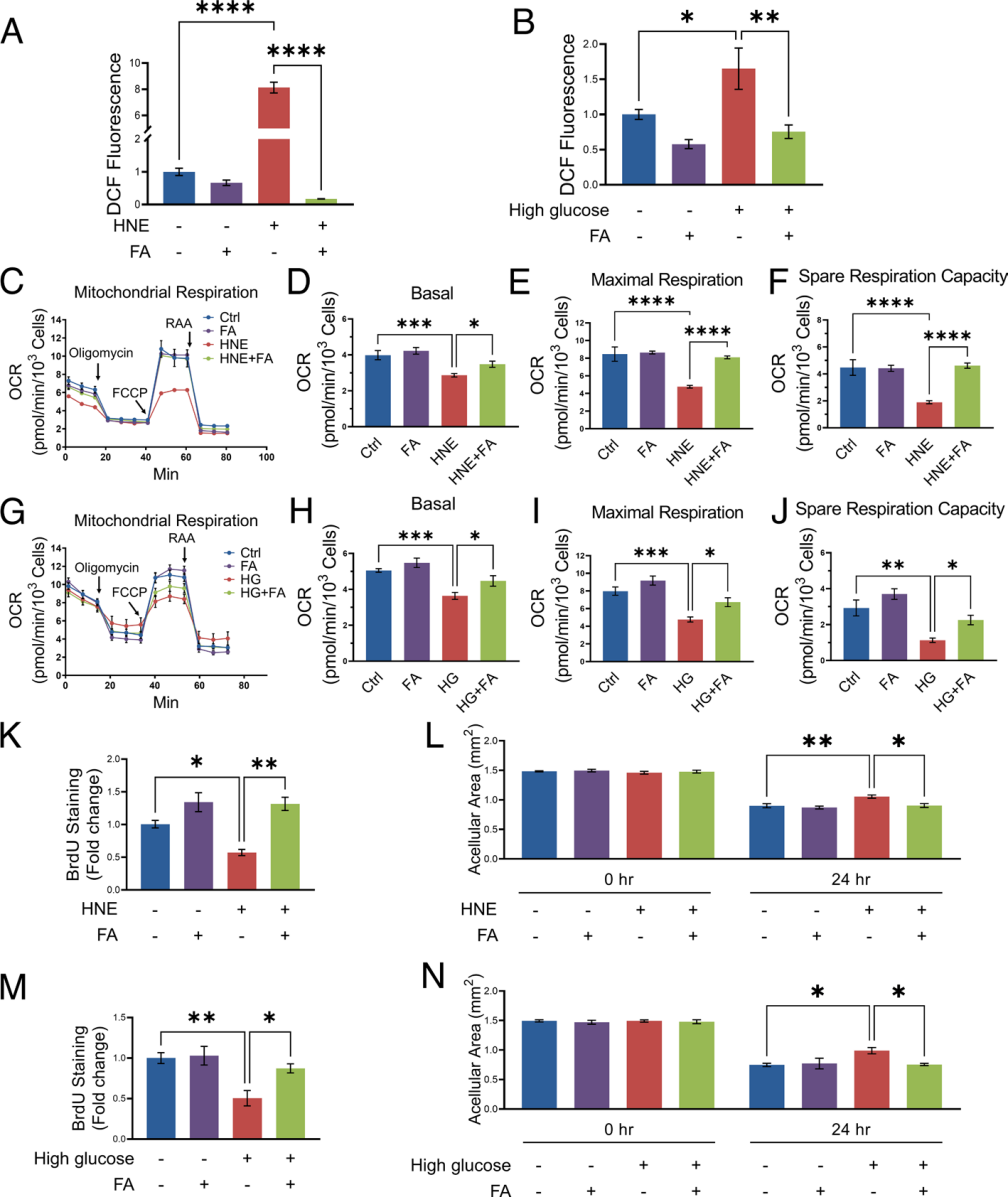
Fenofibric acid treatment ameliorated the mitochondrial dysfunction induced by diabetic stressors, and enhanced proliferation and migration in HCEC. (*A*) The DCF fluorescence signal was measured in HCEC treated with HNE (20 µM) and fenofibric acid (FA, 20 µM) for 24 h (*n* = 5 to 7). (*B*) The DCF fluorescence signal in HCEC treated with high glucose (30 mM) and FA (20 µM) for 4 d (*n* = 8). (*C–F*) Mitochondrial stress test in HCEC treated with HNE (10 µM) and FA (20 µM) for 24 h (*n* = 6 to 8). (*G–J*) Mitochondrial stress test in HCEC exposed to high glucose (30 mM) and FA (20 µM) for 4 d (*n* = 6). (*K*) HCEC proliferation in the presence or absence of HNE (20 µM) and FA (20 µM) for 24 h was measured by Cell Proliferation BrdU ELISA (*n* = 3). (*L*) HCEC were treated with HNE (20 µM) and FA (20 µM) after 100% confluence. Images of each scratch were captured after in vitro “wound” was created at indicated time points. The acellular area was measured by ImageJ (*n* = 6). (*M* and *N*) HCEC proliferation (*n* = 5) and migration (*n* = 5) were measured after the cells were exposed to high glucose (30 mM) and FA (20 µM) for 4 d. Values are mean ± SEM. *P < 0.05; **P < 0.01; ***P < 0.001; ****P < 0.0001.

HNE- and high-glucose–treated HCEC showed an increased production of reactive oxygen species (ROS), which was attenuated by fenofibric acid (Fig. 6 *A* and *B*). The mitochondria stress assay showed that fenofibric acid prevented the decreases of basal, maximal respiration and spare respiratory capacity induced by HNE or high glucose in HCEC (Fig. 6 *C*–*J*).

To establish the direct effect of fenofibric acid on corneal wound healing, we evaluated the proliferation and migration of primary HCEC stressed with HNE and treated with fenofibric acid. As shown in Fig. 6 *K* and *L*, HNE suppressed HCEC proliferation and migration, which was significantly alleviated by fenofibric acid treatment. Similarly, high glucose suppressed HCEC proliferation and migration, which was attenuated by fenofibric acid treatment (Fig. 6 *M* and *N*).

### SiRNA-Mediated Knockdown of PPARα Aggravated Mitochondrial Dysfunction in Primary HCEC.

**Fig. 7.**
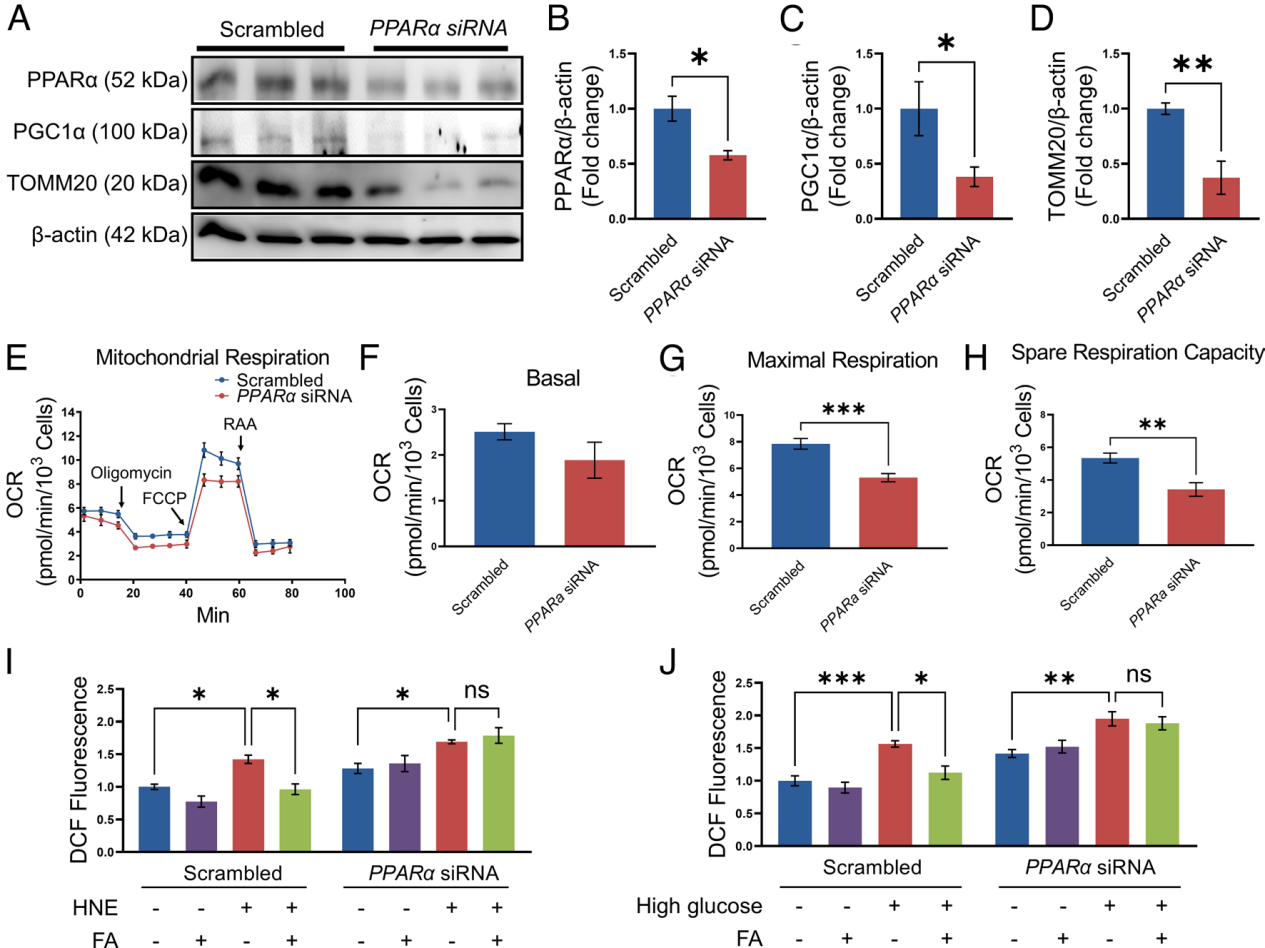
siRNA-mediated knockdown of *PPARα* impaired mitochondrial function in primary HCEC. (*A–D*) Western blot analysis of PPARα, PGC-1α, TOMM20, and β-actin in HCEC transfected with scrambled siRNA or *PPARα* siRNA for 72 h. Protein levels of PPARα (*B*), PGC-1α (*C*), and TOMM20 (*D*) in panel *A* were quantified by densitometry (*n* = 3). (*E–H*) Mitochondrial stress test in the HCEC transfected with siRNA against *PPARα* for 72 h, with scrambled siRNA as control (*n* = 6). (*I*) ROS production was measured in HCEC transfected with scrambled siRNA or *PPARα* siRNA for 72 h and then exposed to HNE (20 µM) and fenofibric acid (FA, 20 µM) for 6 h (*n* = 5 to 6). (*J*) After transfection with scrambled siRNA or *PPARα* siRNA, HCEC were exposed to high glucose (30 mM) and FA (20 µM) for 4 d and the ROS production was measured (*n* = 7). All values are mean ± SEM. *P < 0.05; **P < 0.01; ***P < 0.001; ns, nonsignificant.

To further study the effect of downregulation of PPARα expression on mitochondrial function, HCEC was transfected with a siRNA specific for *PPARα*. Western blotting results confirmed that compared with the scrambled siRNA control group, expression of PPARα was significantly decreased in the cells transfected with the *PPARα* siRNA (Fig. 7 *A* and *B*). Knockdown of *PPARα* suppressed the expression of PGC-1α and TOMM20 in HCEC (Fig. 7 *C* and *D*). The mitochondria stress assay showed that siRNA-mediated knockdown of PPARα decreased maximal respiration and spare respiratory capacity in primary HCEC (Fig. 7 *E*–*H*).

Fenofibric acid treatment significantly attenuated HNE- and high-glucose–induced ROS generation in scrambled siRNA-transfected HCEC. However, the effect of fenofibric acid on ROS production was impeded by the siRNA knockdown of *PPARα*, suggesting a PPARα-dependent mechanism (Fig. 7 *I* and *J*).

## Discussion

Accumulating evidence suggests that mitochondrial dysfunction plays a key role in the pathophysiology of diabetic complications (9–11, 31, 32). Corneal wound healing involves epithelial cell proliferation and migration and requires ATP production. In this study, we found that human corneal epithelial cells use mitochondrial oxidation as a major source of ATP, while corneal stromal fibroblasts predominantly use glycolysis. We also demonstrated declined mitochondrial function in the live corneal punch biopsies of both type 1 and type 2 diabetic mouse models. Furthermore, the present study demonstrated that activation of PPARα alleviated mitochondrial dysfunction and wound healing delay in the diabetic cornea. Consistently, conditional KO of *PPARα* in corneal epithelial cells impaired, while transgenic overexpression of *PPARα* in the corneal epithelium improved mitochondrial function and wound healing in the cornea. These findings suggest that mitochondrial dysfunction plays a key role in deficiency of corneal wound healing in diabetes, and PPARα is an important regulator of mitochondrial function in the corneal epithelium.

Glycolysis and oxidative phosphorylation are the main sources of ATP production. Only a few early documented studies compared metabolic profiles of the corneal epithelium and stroma (33, 34). It was reported by Langham in 1954, that the acid accumulation rate in the rabbit corneal epithelial layer decreases greatly in the presence of oxygen, while in the stroma the acid production rate is barely changed between the anaerobic and aerobic environment, suggesting that epithelium prefers aerobic environment than stroma (33). In 1985, Greiner et. al found that phosphates associated with energy metabolism were primarily in the corneal epithelium using phosphorus-31 NMR (34) and suggested that high-energy phosphate biosynthesis occurs in the corneal epithelium. There was no recent study regarding human corneal metabolic profiles, leaving a major knowledge gap. Here, we for the first time compared the metabolic profiles of primary human epithelial cells with those of human stromal fibroblasts side by side. To exclude individual differences from human donors, we used primary corneal epithelial cells and stromal fibroblasts from six different donors and provided the direct evidence showing that mitochondrial oxidation is a major energy source in human epithelial cells by measuring the real-time ATP production. Under the same condition, human stromal fibroblasts predominantly use glycolysis to generate ATP. These results demonstrate that human corneal epithelial cells and stromal fibroblasts have different metabolic profiles, suggesting that regulation of mitochondrial function has a significant impact on epithelial function and wound healing.

Previous studies have suggested that the corneal cells have declined mitochondrial function in diabetes. Mussi et al. showed high-glucose culture compromises mitochondrial function in human telomerase-immortalized corneal epithelial cells (35). Aldrich et al. reported that corneal endothelial cells of human donors with advanced diabetes have impaired mitochondrial function as measured by extracellular flux analysis (36). Here, for the first time, we investigated mitochondrial function using live corneal biopsies from both type 1 and type 2 diabetic mice for real-time measurement of mitochondrial function. For the Seahorse analysis, we placed 1.5-mm corneal punches into the plate with the epithelium side on top which is close to the sensor probes of the Seahorse analyzer. Considering that the epithelial cells are more dependent on mitochondria for energy and the epithelium side is closer to the probe, we believe that the OCR measured in corneal biopsies likely reflects the mitochondrial oxidation of the epithelium. The results demonstrated that mitochondrial function was impaired in the corneal epithelium of both type 1 and type 2 diabetic models.

To measure the metabolic activities in isolated corneal epithelium and stroma, we separated the epithelial layer and stroma layer for Seahorse analysis. However, after peeling off the epithelial layer, the mitochondrial function from the isolated epithelial layer or stroma layer became nondetectable by Seahorse assay, suggesting possible damage during the dissection procedure. This represents a limitation of the mitochondrial assay using corneal punches for the Seahorse assay.

To explore the molecular basis for the diabetes-induced mitochondrial function decline in the cornea, we evaluated the role of PPARα. PPARα is a transcription factor that belongs to the nuclear receptor superfamily (37). Upon activation, PPARα heterodimerizes with the Retinoid X Receptor (RXR) and binds to PPAR Response Elements (PPREs) in the promoter regions of target genes including PPARα itself and those involved in many processes such as energy metabolism, oxidative stress, inflammation, circadian rhythm, immune response, mitochondrial genesis, and cell differentiation (12, 38–43). Two independent, prospective clinical studies reported robust therapeutic effects of PPARα agonist fenofibrate on diabetic retinopathy in type 2 diabetic patients (44). Our previous study has shown that diabetes-induced downregulation of PPARα in the retina plays a key pathogenic role in retinal oxidative stress and inflammation in diabetic retinopathy (45). Recently, we demonstrated that PPARα protein levels are decreased in the corneal epithelium from both T1DM and T2DM donors compared to nondiabetic human donors (18). *PPARα^−/−^* mice showed declined corneal nerve densities and increased epithelial lesion in the central cornea (18). It has been reported that PPARα agonist accelerated corneal epithelial healing after alkali injury (46). These observations suggested that PPARα has a role in the maintenance of corneal integrity. However, the physiological function of PPARα in the cornea, especially in the regulation of mitochondria function, has not been previously investigated.

To evaluate the impacts of PPARα downregulation on metabolic profile in the diabetic cornea, we treated diabetic mice with PPARα agonist fenofibrate. Our results showed that fenofibrate treatment ameliorated mitochondrial dysfunction in diabetic corneas. Consistently, fenofibrate treatment also improved wound healing in diabetic corneas. In contrast, *PPARα* KO alone impaired mitochondrial function and delayed corneal wound healing. Global *PPARα* KO has been shown to result in systemic hyperlipidemia (47). To exclude potential impacts secondary to systemic dyslipidemia in global *PPARα* KO, we generated epithelium-specific *PPARα* conditional KO and transgenic mice for this study. Consistent with the observations from the *PPARα^−/−^* mice, epithelium-specific *PPARα* conditional KO mice also showed impaired mitochondrial function and wound healing delay in the cornea. In contrast, transgenic overexpression of *PPARα* in the epithelium alleviated the mitochondrial dysfunction as well as corneal wound healing delay. These results suggested that epithelial PPARα is important for the regulation of mitochondria function in the cornea.

PPARα is known to regulate PGC-1α (48, 49). PGC-1α plays essential roles in glucose/fatty acid metabolism and mitochondria biogenesis (50–52). TOMM20, often used as a surrogate of mitochondria mass, is regulated by PGC-1α (51–55). In this study, we demonstrated that *PPARα* KO mice displayed decreased expression of PGC-1α and TOMM20 in the cornea. To further define the direct effect of PPARα, we treated diabetic mice and cultured primary HCEC with PPARα agonist, fenofibrate, or fenofibric acid. Activation of PPARα restored the expression of PGC-1α and TOMM20 in the cornea of diabetic mice. Exposure of primary HCEC to diabetic stressors downregulated the expression of PPARα and impaired ATP production from mitochondria, while glycolysis remained intact. Activation of PPARα using fenofibric acid increased the expression of PGC-1α and TOMM20 and attenuated the decline of proliferation and migration of epithelial cells under diabetic stress. Consistent with the in vivo observation in global *PPARα* KO and conditional KO mice, siRNA-mediated knockdown of *PPARα* alone downregulated the expression of PGC-1α and TOMM20 in these cells. Further, mitochondrial fragmentation and mitochondrial membrane electric potential measurements support that diabetic stressor impairs integrity of mitochondria, which can be attenuated by PPARα agonist. Although fenofibric acid is a PPARα agonist, off-target effects were reported (56–59). Here, we showed that the beneficial effect of fenofibric acid on ROS production was impeded by the PPARα siRNA knockdown, suggesting that fenofibric acid ameliorated epithelial cell mitochondrial function through a PPARα-dependent mechanism. Therefore, it is plausible to suggest that PPARα signaling in epithelial cells may represent an important regulatory mechanism for mitochondrial metabolism and corneal wound healing.

Recent studies have shown that disturbed epithelial–neural–immune cell interactions are a major cause of diabetic neurotrophic keratopathy (5). Our previous study showed that PPARα protects corneal nerve against DM (18). The present study demonstrated that PPARα also promotes corneal epithelial cell proliferation and migration likely through protection of the metabolic function. The regulation of epithelial cell metabolism may also improve neurotrophic environment in the cornea and thus contributes to nerve protection, which remains to be further investigated.

In conclusion, the present study identified for the first time that PPAR*α* serves as an important regulator of mitochondrial function in the corneal epithelium, and diabetes-induced PPARα downregulation plays a pathogenic role in wound healing deficiency in the diabetic cornea. PPARα has potential to become a new therapeutic target, and fenofibrate may have therapeutic potential in diabetic keratopathy.

## Materials and Methods

### Ethical Approval and Informed Consent.

The study adhered to the tenets of the Declaration of Helsinki and was performed with the Institutional Review Board (IRB) approval from the University of Oklahoma Health Sciences Center (IRB protocol #3450). Donor eyes from patients with diabetes and nondiabetic controls were obtained from Lions Gift of Sight Eye Bank (Saint Paul, MN). All methods were performed in accordance with federal and institutional guidelines and all human samples were deidentified prior to analysis.

### PPARα RNAscope In Situ Hybridization.

Human donor eyes dissected within 12 h postmortem were immediately preserved in Davidson’s fixation solution for 24 h and then transferred to 10% buffered formalin for storage and paraffin section. Human PPARα fluorescent in situ hybridization was performed using the RNAscope Multiplex Fluorescent V2 Assay kit (Advanced Cell Diagnostics #323110 and #445201; Santa Ana, CA) according to the manufacturer’s instruction. Negative control assays were conducted using a 3-plex negative control probe provided by the manufacturer (Advanced Cell Diagnostics #320871). In situ hybridization was followed by DAPI staining, and then, fluorescent signals were photographed under an Olympus laser scanning confocal microscope (FV1200; Bartlett, TN). The total number of PPARα mRNA puncta in the corneal epithelium was counted by Image J (NIH, Bethesda, MD) and normalized by the number of nuclei to quantify the expression of PPARα mRNA.

### Animals.

Male *PPARα^−/−^* mice (20-wk-old), wild-type (WT) C57BL/6J mice (8-wk-old), Akita (*Ins2^akita^*) mice, *db/db* (BKS.Cg-*Lepr^db^*/J) mice, and their heterozygous littermates in the C57BLKS/J background were purchased from Jackson Laboratories (Bar Harbor, ME). All experiments were performed following the guidelines of the Association for Research in Vision and Ophthalmology (ARVO) Statement for the Use of Animals in Ophthalmic and Vision Research and approved by the Institutional Animal Care and Use Committee of the University of Oklahoma Health Sciences Center. In all procedures, 50 mg/kg ketamine hydrochloride mixed with 5 mg/kg xylazine (Vedco) was used for intraperitoneal injection to anesthetize the mice.

### Corneal Epithelium-Specific PPARα Transgenic Mice and PPARα Conditional KO Mice.

Corneal epithelium-specific Cre transgenic (*Krt12-Cre*) mice were purchased from Jackson Laboratories (stock#023055). PPARα transgenic flox/flox (PCTG) mice which express PPARα under the chicken β-actin promoter upon removal of a floxed stop cassette by desired tissue-specific Cre recombinase were generated through a contract service with Cyagen Biosciences (Santa Clara, CA) following a documented strategy (60). *Krt12-Cre* mice were crossed with PCTG mice to remove of the floxed stop cassette for the generation of *PPARα^ECTg^* mice, which overexpress the PPARα transgene in the corneal epithelium. Overexpression of PPARα in the corneas of *PPARα^ECTg^* mice was verified by immunohistochemistry, in comparison with *Krt12-Cre* mice. *PPARα^flox/flox^* mice in which *PPARα* exon 4 was flanked with loxP sites were generated through a contracted service with Ingenious Targeting Laboratory (Ronkonkoma, NY). To generate corneal epithelium-specific *PPARα* knockout mice (*PPARα^ECKO^*), *PPARα^flox/flox^* mice were crossbred with *Krt12-Cre* mice, and the KO efficiency of *PPARα* was verified by immunohistochemistry in the corneas of *PPARα^ECKO^* mice relative to those in *Krt12-Cre* mice.

### STZ-Induced Diabetic Mouse Model.

As described previously, to induce diabetes, male WT mice (8-wk-old) received daily intraperitoneal injections of STZ at a dose of 55 mg/kg for five consecutive days (20). Mice with blood glucose levels higher than 350 mg/dL were defined as diabetic animals. The diabetic mice were randomly assigned into two groups: one fed special chow containing 0.014% fenofibrate (LabDiet 5053; TestDiet) and the other fed regular chow as control for 12 wk.

### Corneal Epithelial Debridement Wound Healing Model.

The corneal wound was induced following a documented protocol (61). After mice were anesthetized, an Algerbrush II Corneal Rust Ring Remover (Alloy Medical) was used to create a circular wound of 2 mm diameter on the central corneal epithelium. The abraded region was stained with 0.1% sodium fluorescein and photographed daily with Micron IV (Phoenix Technology Group). The wound area was quantified with ImageJ software.

### Immunohistochemistry of Mouse Corneas.

The mouse eyeballs were fixed in Davidson’s fixation solution for 48 h for the paraffin section. Following the antigen retrieval with sodium citrate buffer (10 mM sodium citrate, 0.05% Tween 20, pH 6.0) in a steam bath, three washes with phosphate-buffered saline (PBS) and blocking with 5% bovine serum albumin (BSA) in PBS, the sections were incubated with anti-PPARα (Novus #NB600-636; Centennial, CO), anti-TOMM20 (abcam #ab186735; Waltham, MA), or anti-nitrotyrosine (abcam #ab61392) antibodies overnight. After three washes with PBS, the slides were incubated with Alexa Fluor 488 AffiniPure donkey anti-rabbit IgG (Jackson ImmunoResearch #711-545-152; West Grove, PA) or Alexa Fluor 594 AffiniPure donkey anti-mouse IgG (Jackson ImmunoResearch #715-585-150). The slides were mounted with Vectashield mounting buffer containing DAPI (Vector Laboratories #H-1200; Newark, CA) and photographed under a Zeiss Microscope (Observer Z1; Pleasanton, CA).

### Real-Time ATP Rate Assay in Cultured Cells.

Primary human corneal stromal fibroblasts were isolated from six healthy donors (two females and four males whose average age was 41.2 y) by explant culture in EMEM (ATCC #30-2003; Manassas, VA) supplemented with 10% FBS and 1% antibiotic-antimycotic. Upon cell expansion, the cells were passaged and maintained in the same medium for subculture. Primary human corneal epithelial cells (HCEC) from six donors (two females and four males, average age 33.5 y) were purchased from ATCC (PCS-700-010) and cultured in medium containing 6.2 mM glucose recommended by the manufacturer (PCS-700-030 and PCS-700-040). Analysis of the metabolic profile in the cells between passages 3 to 6 was performed using a Seahorse XFe96 Flux Analyzer (Agilent Technologies). The cells were seeded in a 96-well Seahorse microplate at a density of 5 × 10^3^ cells/well, and specific compounds including oligomycin (1.5 μM) and a combination of rotenone and antimycin A (RAA, 0.5 μM) were prepared in the cartridge and sequentially injected to measure ATP generation.

### Mitochondrial Stress Test.

Mice were euthanized with overdose ketamine/xylazine, the whole corneas were isolated, and a 1.5-mm punch biopsy was prepared from the central cornea and loaded into a 96-well spheroid plate with the epithelium side up. Corneal punches were incubated in Seahorse XF base medium (Agilent # 103335-100) supplemented with 10 mM glucose, 1 mM pyruvate, and 2 mM glutamine. OCR was measured following the protocol for the mitochondrial stress assay using the Seahorse XFe96 analyzer with sequential injections of oligomycin (1.5 μM), carbonyl cyanide 4-(trifluoromethoxy) phenylhydrazone (FCCP, 2.0 μM), and RAA (0.5 μM). Basal respiration was calculated by subtracting nonmitochondrial respiration from the basal rate prior to the injection of oligomycin. Maximal respiration was calculated by subtracting nonmitochondrial respiration from the rate after injection of FCCP.

Primary HCEC were cultured at a density of 5 × 10^3^ cells/well in XF 96-well microplates with/without HNE (EMD Millipore #393204; Burlington, MA) for 24 h. Hyperglycemic stress was induced by the addition of 23.8 mM D-glucose (Sigma Aldrich #G7528; St. Louis, MO) for 4 d, and the same concentration of L-glucose (Sigma Aldrich #G5500) was used as a control. Real-time measurement of OCR was recorded with the injection of 1.5 µM Oligomycin, 2.0 µM FCCP, and 0.5 µM RAA.

### ROS Assay.

The production of ROS in HCEC was measured using CM-H_2_DCFDA (Invitrogen #C6827; Waltham, MA) following the protocol of the manufacturer. Following the treatment of the conditioned medium containing HNE/high glucose and fenofibric acid, the cells were washed with PBS and then incubated with CM-H_2_DCFDA (1:5,000 dilution) at 37 °C for 30 min. ROS levels were determined by measuring the fluorescence intensity at an excitation wavelength of 483 nm and at an emission wavelength of 530 nm using the Wallac 1420 microplate reader (PerkinElmer Life and Analytical Sciences, Shelton, CT). ROS concentrations were quantified by normalization of the ROS level to the cell protein concentration.

### Western Blot Analysis.

Western blot analysis was performed as described previously (20). The equal amount of protein was resolved by SDS-PAGE and immunoblotted with primary antibodies for PPARα (Novus #NB600-636), TOMM20 (abcam #ab186735), PGC-1α (Novus #NBP1-04676), and β-actin (Sigma-Aldrich #A5441). Primary antibodies were then detected with HRP-conjugated secondary antibodies (Vector Laboratory #PI-1000 & PI-2000), and the band intensity was semiquantified by densitometry using ImageJ software and normalized by β-actin levels.

### Staining of Mitochondria and Manual Scoring of Mitochondrial Morphology.

HCEC were seeded in 8-chamber culture slides (FALCON #354108; Glendadle, AZ). After treatment, the cells were fixed in 4% PFA for 20 min, washed with PBS containing 0.3% Tween 20, and 0.3% Triton-X-100 three times, then incubated with TOMM20 antibody (abcam #ab186735) at 4 °C overnight. Alexa Fluor 488 AffiniPure donkey anti-rabbit IgG (Jackson ImmunoResearch #711-545-152) was applied for 2 h, and the slides were mounted with Vectashield mounting buffer containing DAPI (Vector Laboratories #H-1200). The cell images were captured under a Zeiss Observer Z1 Microscope and manually classified as “fragmented” if >50% of mitochondrial area consisting of punctiform mitochondria as described previously (62, 63).

### Mitochondrial DNA Copy Number Measurement.

Total DNA was extracted from HCEC using a DNA extraction kit (Zymo Research #D7003; Irvine, CA). The mtDNA levels were measured by qPCR and normalized by nuclear DNA levels as previously described (64). The mtDNA primers used were forward 1 (5′-CACCCAAGAACAGGGTTTGT-3′) and reverse 1 (5′-TGGCCATGGGTATGTTGTTA-3′) and nuclear DNA primers were forward 1 (5′-TGCTGTCTCCATGTTTGATGTATCT-3′) and reverse 1 (5′-TCTCTGCTCCCCACCTCTAAGT-3′).

### Mitochondrial Membrane Electric Potential (Δψ_m_).

Δψ_m_ of HCEC was measured using potentiometric dye JC-1 (Invitrogen #T3168) following the protocol of the manufacturer. HCEC were cultured at the condition described above. The cells were stained with 2 µM JC-1 for 30 min and then evaluated for mean cell fluorescence by flow cytometry BD LSRFortessa X-20 Analyzer (Becton Dickinson).

### Bromodeoxyuridine (BrdU) Cell Proliferation.

The BrdU Cell Proliferation ELISA Kit (ab126556) from Abcam was used to access cell proliferation rate. Briefly, after incubation with BrdU for 24 h, the cells were fixed for 30 min. Then, the cells were incubated with an anti-BrdU antibody for 1 h. Next, peroxidase goat anti-mouse IgG and the HRP substrate were added to the cells and incubated for 30 min. The absorbance was measured at 450 nm using a microplate reader.

### Cell Migration Assay.

After the cells reached 100% confluence in 12-well plates, a 200-µL sterile pipette tip was used to make a scratch as documented previously (65). The cells were washed twice with PBS to remove floating cells and cultured in treatment media. The scratch acellular area was photographed under Cytation 1 Cell Imaging Multi-Mode Reader (BioTek) at two preselected time points (0 and 24 h). The acellular area was measured using the ImageJ software.

### Transfection of HCEC with siRNA.

PPARα expression was knocked down in HCEC by transfection with the SMARTpool human *PPARα* siRNA (Dharmacon #D-001206-13-20; Boulder, CO) with nontargeting siRNA SMARTpool as control (Dharmacon #D-001810-10-50) using HiPerFect transfection reagent (QIAGEN #301704; Germantown, MD) following the manufacturer’s instruction.

### Statistical Analysis.

Data were presented as mean ± SEM and analyzed by unpaired Student’s *t* test for two groups and ANOVA for more than two groups. *P* < 0.05 was considered statistically significant.

## Data Availability

All data are contained within this article. Noncommercial reagents described in this manuscript are available upon request.
